# Analysis of expression, epigenetic, and genetic changes of HNF1B in 130 kidney tumours

**DOI:** 10.1038/s41598-020-74059-z

**Published:** 2020-10-13

**Authors:** Michaela Bártů, Jan Hojný, Nikola Hájková, Romana Michálková, Eva Krkavcová, Ladislav Hadravský, Lenka Kleissnerová, Quang Hiep Bui, Ivana Stružinská, Kristýna Němejcová, Otakar Čapoun, Monika Šlemendová, Pavel Dundr

**Affiliations:** 1 Institute of Pathology, First Faculty of Medicine, Charles University and General University Hospital in Prague, Studnickova 2, 12800 Prague 2, Czech Republic; 2 Department of Urology, General University Hospital in Prague, 12808 Prague, Czech Republic

**Keywords:** Cancer, Genetics, Molecular biology, Biomarkers, Molecular medicine, Nephrology, Pathogenesis

## Abstract

Hepatocyte nuclear factor 1 beta (HNF1B) is a transcription factor which plays a crucial role in nephronogenesis, and its germline mutations have been associated with kidney developmental disorders. However, the effects of HNF1B somatic exonic mutations and its role in the pathogenesis of kidney tumours has not yet been elucidated. Depending on the type of the tumour HNF1B may act as a tumour suppressor or oncogene, although the exact mechanism by which HNF1B participates in the process of cancerogenesis is unknown. Using an immunohistochemical approach, and methylation and mutation analysis, we have investigated the expression, epigenetic, and genetic changes of HNF1B in 130 cases of renal tumours (121 renal cell carcinomas, 9 oncocytomas). In the subset of clear cell renal cell carcinoma (ccRCC), decreased HNF1B expression was associated with a higher tumour grade and higher T stage. The mutation analysis revealed no mutations in the analysed samples. Promoter methylation was detected in two ccRCCs and one oncocytoma. The results of our work on a limited sample set suggest that while in papillary renal cell carcinoma HNF1B functions as an oncogene, in ccRCC and chRCC it may act in a tumour suppressive fashion.

## Introduction

Hepatocyte nuclear factor 1 beta (*HNF1B*, previously also known as *TCF2*) is a tissue-specific, developmentally regulated transcription factor which is crucial for the embryonic development of organs derived from ventral endoderm, including the kidneys, pancreas, gastrointestinal system, liver, biliary tract and genital tract^1, 2^. *HNF1B* is located at chromosome 17q12 and plays a crucial role in the early stages of nephron development, as it is required for the activation of a segment-specific gene expression program responsible for the development of nephrons^3^. Heterozygous germline mutations of *HNF1B* are the most common monogenic cause of developmental kidney disease and are associated with a wide variety of congenital kidney malformations, which ultimately lead to chronic renal disease in the afflicted individuals^4–7^. The disease phenotype especially includes Renal Cysts and Diabetes Syndrome (RCAD, OMIM #137920), which may also be associated with the dysfunction of pancreatic β-cells, leading to a subtype of diabetes mellitus (maturity-onset diabetes of the young, MODY5)^8–11^.

To date, more than 100 different germline *HNF1B* mutations scattered across the gene have been reported in literature, all presenting with a varied range of phenotypes of associated kidney, urogenital tract, and pancreas disorders^8, 12^. They comprise mainly base substitutions, small insertion-deletions, or whole-gene deletions which are inherited in an autosomal dominant fashion, although up to 50% of them arise de novo^5, 10, 13^.

While the role of HNF1B in developmental kidney anomalies has already been well established, its role in the pathogenesis of kidney tumours has not yet been elucidated, despite there being an increasing number of studies pointing to its involvement in the pathogenesis of several types of cancers, including kidney cancer.

As yet, the effects of somatic exonic mutations of *HNF1B* on cancerogenesis and tumour development have not been fully explained, although there are several genome-wide association studies (GWAS) which have identified several single nucleotide polymorphisms (SNPs) in the *HNF1B* gene as associated with either an increased or a decreased risk of prostate cancer^14, 15^, endometrial cancer^16, 17^, and even kidney cancer^18^. According to literature, HNF1B protein may act as either a protooncogene or a tumour suppressor depending on the type of tumour and its histogenesis^15, 19, 20^. Nonetheless, the precise mechanism by which HNF1B participates in the process of cancerogenesis is unknown, and probably differs in different types of tumours.

Although HNF1B plays such a profound role in the development of the kidneys, knowledge about its involvement in the pathogenesis of kidney tumours is sparse, with only a handful of published studies focusing on the relationship between HNF1B and malignant or benign kidney lesions^3, 21–23^. Malignant kidney tumours make up to 2% of all cancers and, until recently, their incidence had been constantly rising^24, 25^. The most common histologic subtype is clear cell renal cell carcinoma (ccRCC, 70–80%), followed by papillary renal cell carcinoma (papRCC, 10–20%), and chromophobe renal cell carcinoma (chRCC, 5%)^26^. The landscape of molecular aberrations implicated in the pathogenesis of kidney tumours is very diverse and includes both chromosomal gains and losses, with a number of genes (such as *VHL, PBRM1, SETD2, BAP1, MET, FH*) already being established as key players in the development of RCC^27^. The role of others, including HNF1B, still remains to be elucidated.

Therefore, our aim was to perform a comprehensive analysis of the expression, epigenetic, and genetic changes of HNF1B in kidney tumours, and to further investigate its potential differential diagnostic, prognostic and therapeutic value, mainly at the level of somatic changes.

## Material and methods

### Samples

The study was performed on formalin-fixed, paraffin-embedded (FFPE) tissue blocks and, where available, the corresponding fresh-frozen tissue (FT) stored in the RNAlater stabilization solution (Qiagen) at − 80 °C according to the manufacturer´s protocol (Stabilization of RNA in Harvested Animal Tissues; Qiagen), as described in our previous work^28^. The FFPE tissue blocks were sourced from the archives of our department and the corresponding FT samples were provided by the Bank of Biological Material (BBM) of the First Faculty of Medicine, Charles University in Prague.

A total of 130 FFPE tissue samples (Table 1) were used for the immunohistochemical analysis, consisting of 93 cases of clear cell renal cell carcinoma, 17 cases of papillary renal cell carcinoma, 11 cases of chromophobe renal cell carcinoma, and 9 cases of renal oncocytoma (RO). Out of these 130 cases there were 56 cases (42 ccRCC, 2 papRCC, 3 chrRCC, 9 oncocytomas) with available corresponding FT sample pairs (tumour and in some cases non-tumour tissue), which were used for DNA isolation and subsequently mutation and promoter methylation analysis. The clinicopathologic characteristics (analysed for the ccRCC subset of samples) are summarized in Table 2. The main evaluated parameters included tumour stage (TNM) and grade where applicable (according to the 4th Edition of WHO Classification of Tumours of the Urinary System and Male Genital Organs and International Society of Urological Pathology)^29^, lymphovascular invasion, presence of metastases, recurrence, gender, and age at the time of diagnosis. The hematoxylin and eosin-stained slides of all of the selected kidney tumour samples were reviewed and suitable tumour areas were marked for the removal of individual tissue cores, which were used for the construction of tissue microarrays (TMAs). From each tumour donor block two tissue cores (each 2.0 mm in diameter) were drilled using the tissue microarray instrument TMA Master (3DHISTECH Ltd., Budapest, Hungary) and the evaluation of the studied tumours was performed with the use of TMAs.

**Table 1:** Association of HNF1B expression and type of diagnosis, based on 130 cases of renal cell carcinoma.

Group	N	H-score mean	H-score median	*p*-value^a^	Categorized H-score			
					Group 1	Group 2	Group 3	*p*-value^b^
**Diagnosis**								
ccRCC	93	201.3	250	** < 0.001**	22	18	53	**0.004**
chRCC	11	35.5	0		9	2	0	
papRCC	17	201.8	270		5	3	9	
RO	9	162.2	170		3	3	3	

Statistically significant results (*p*-value < 0.05) are highlighted in bold.

*ccRCC* clear cell renal cell carcinoma, *chRCC* chromophobe renal cell carcinoma, *papRCC* papillary renal cell carcinoma, *RO* renal oncocytoma.

^a^*p* values are based on Mann–Whitney U-test or Kruskal–Wallis H-test.

^b^*p* values are based on Pearson chi-squared test (categorized expression).

**Table 2:** Association of HNF1B expression and clinicopathological characteristics, based on 93 cases of ccRCC.

Characteristic	Group	N	H-score mean	H-score median	*p*-value^a^	Categorized H-score			
						Group 1	Group 2	Group 3	*p*-value^b^
Gender					0.617				0.399
	Male	54	198.2	240		12	13	29	
	Female	39	205.8	250		10	5	24	
Age (mean = 63, median = 65)					0.849				0.414
	˂ 65	44	198.9	240		8	8	28	
	≥ 65	49	203.5	260		14	10	25	
pT classification					0.094				0.360
	pT1	70	212.4	265		15	11	44	
	pT2	6	141.7	145		2	2	2	
	pT3	17	177.1	170		5	5	7	
Grade					**0.002**				**0.012**
	G1	22	255.5	300		2	2	18	
	G2	53	196.1	240		14	9	30	
	G3	13	167.7	170		3	6	4	
	G4	5	106.0	50		3	1	1	
Lymphovascular invasion*					0.264				0.558
	Yes	15	177.3	200		5	3	7	
	No	75	207.1	260		16	14	45	
Metastasis*					0.121				0.135
	Yes	10	158.0	140		3	4	3	
	No	81	207.8	250		18	14	49	
Recurrence*					0.458				0.899
	Yes	8	183.8	210		2	2	4	
	No	83	204.1	250		19	16	48	
rs4430796*					0.727				0.951
	Yes	31	207.7	270		6	2	21	
	No	8	276.3	295		0	1	5	
rs757210*					0.384				0.528
	Yes	36	216.1	275		0	0	3	
	No	3	290.0	290		6	3	23	

Statistically significant results (*p*-value < 0.05) are highlighted in bold.

^a^*p* values are based on Mann–Whitney U-test or Kruskal–Wallis H-test.

^b^*p* values are based on Pearson chi-squared test (categorized expression).

*Data are not available for all cases.

### Ethical approval

The study has been approved by the Ethics Committee of the General University Hospital in Prague in compliance with the Helsinki Declaration (ethical approval number 41/16 Grant VES 2017 AZV VFN). The Ethics Committee did not require the procurement of informed consent, given that according to the Czech Law (Act. no. 373/11, and its amendment Act no. 202/17) it is not necessary to provide informed consent in fully anonymized studies.

### DNA isolation and quality control

The DNA isolation process was performed as described in our previously published study focusing on the problematics of HNF1B^28^. All of the analysed DNA was obtained from FT samples (stored in RNAlater), which were first thawed, and then homogenized (10–30 mg of the tissue) using MagNA Lyser Green Beads tubes in a MagNA Lyser Instrument (Roche) in the presence of 600 µl of RLT Plus buffer (Qiagen) with 6 µl of 14.3 M 2-mercaptoethanol (Sigma-Aldrich). The total DNAs and RNAs were isolated according to the Simultaneous Purification of Genomic DNA and Total RNA from Animal Tissues protocol by using an AllPrep DNA/RNA Mini kit (Qiagen). The quantification of the isolated DNA samples was carried out with the use of NanoDrop 2000 (Thermo Fisher).

### Immunohistochemical analysis

Using the constructed TMAs, the immunohistochemical (IHC) analysis was performed on all of the kidney tumour samples using the standard 4 μm thick sections of FFPE tissue, in accordance with methodology described in our previous work^28^. The slides were stained with the automated staining instrument Ventana BenchMark ULTRA (Roche, Basel, Switzerland) with a rabbit antibody against the HNF1B protein (polyclonal, dilution 1:500, product no. HPA002083, Sigma-Aldrich, Prestige Antibodies, St. Louis, United States). The heat induced epitope retrieval with a citrate buffer (pH 6.0) was used for pre-treatment. The detection of the primary antibody was visualized with the OptiView DAB IHC Detection Kit (Ventana, Roche). Only the nuclear staining was evaluated as positive and the nuclear expression of HNF1B was double-blindly evaluated by two independent pathologists.

The immunohistochemical results were evaluated first according to the overall percentage of positive cells (0–100%), with the positive cells then being stratified and assessed also semi-quantitatively, using the H-score method previously described by others^30^. The H-score assessment method is based on determining the percentage of positive cells based on the level of staining intensity (1 + for weak intensity, 2 + for moderate and 3 + for strong intensity). The final H-score for each case is then obtained through the following formula by adding the multiplication of the different staining intensities: [1 × (% of cells 1 +) + 2 × (% of cells 2 +) + 3 × (% of cells 3 +)]. The final H-score value then ranges between 0 and 300.

In each of the evaluated IHC stained slides of tumour tissue cores the presence of internal negative and positive controls was also assessed. The positive control was represented by the staining of normal, non-neoplastic tubular epithelia present in each IHC run, while the negative control was provided in the form of a lack of staining in other structures (such as the connective tissue, smooth muscle, and adipose tissue).

### Statistical analyses

All of the statistical analyses were performed with the use of the Statistica software (StatSoft, Inc., Tulsa, OK). The association between HNF1B expression (using H-score as a continuous dependent variable) and clinicopathological characteristics (categorical variables) was analysed with the nonparametric ANOVA approach. Based on the number of categories either the Mann–Whitney U-test (two categories) or the Kruskall-Walis H-test (three and more categories) were used. In case of the Kruskall-Walis H-test, *post-hoc* tests (pairwise multiple comparison of mean ranks) were conducted to determine which groups differed from each other. When evaluating the effect of independent clinicopathological characteristics on the categorized H-score, the Pearson chi-square test was used. Given that in the largest subset of samples (ccRCC) there were 8 / 93 cases of local or distant recurrence, survival analyses would not have yielded relevant results and therefore were not plotted. For the purposes of the chi-squared tests the H-score was categorized into three groups (group 1: H-score 0–100; group 2: H-score 101–200; group 3: H-score 201–300). All tests were two-sided and a *p*-value of less than 0.05 was considered as significant.

### Genetic and epigenetic analysis

The molecular analyses consisted of DNA mutation analysis of the coding parts of the *HNF1B* exons with adjacent intronic sequences (± 15 bp) and epigenetic analysis of CpG methylation in the region of the *HNF1B* promoter. The *HNF1B* mutation analysis of high-quality FT DNA samples (56 tumour and 42 paired non-tumour tissues) was performed by in-house 2-step polymerase chain reaction (PCR) amplicon next-generation sequencing (NGS). Of the 56 tumour samples, three did not meet the sufficient concentration requirements and therefore in their case the mutation analysis was not performed.

#### Amplicon NGS preparation and sequencing

The in-house 2-step PCR amplicon approach was carried out with the use of the 15 primer pairs (list of primers is provided in Supplementary Table 1) with universal adaptor sequences, which were designed to fit the specific *HNF1B* gene regions in the first PCR step. The selected *HNF1B* gene regions also included the deep intronic regions containing the rs7527210 and rs4430796 variant sites. The second PCR step was carried out with the use of a universal primer pair containing Illumina sequencing adaptor sequences (Supplementary Table 1). The first PCR step which covered the *HNF1B* target regions was performed in two separate multiplex reactions. In order to eliminate undesirable primer interactions each reaction included a different primer pair set (Supplementary Table 1). Both PCR reactions were amplified using the FastStart High Fidelity PCR System (Roche) according to the recommended standard PCR procedure (FastStart High Fidelity PCR System; Roche) in 20 µl reactions according to the following PCR protocol: 2 min—95 °C; 10 cycles of 15 s—95 °C, 20 s—62 °C and 30 s—72 °C (all steps with ramping temperature 2 °C/s) and then 20 cycles of 15 s—95 °C, 20 s—62 °C and 30 s—72 °C (standard ramping temperature 4 °C/s). Following the first PCR step, 10 µl of both of the first PCR reactions were equimolarly mixed and purified with the AMPure XP system (0.8 ×; Beckmann Coulter). The second PCR step was then employed in order to amplify the purified PCR product, consisting of 10 cycles using the same protocol, with a standard ramping rate and different primers (the universal primer pair with Illumina adaptor sequences was used).

When the second PCR step was completed, the concentrations of the PCR products were measured with the use of the Qubit fluorimeter (Thermo Fisher) and then equimolarly mixed to create one sequencing library. The prepared sequencing library was then purified using the AMPure XP system (0.8 ×; Beckmann Coulter) and measured for concentration (Qubit) and for fragment length using the High Sensitivity NGS Fragment Analysis kit on the Fragment Analyzer (AATI). This amplicon library was then sequenced together with different (capture) libraries in order to increase sequencing heterogeneity. The sequencing was performed either using 50 samples, which were sequenced in one amplicon library by the MiSeq 300 cycles v2 kit or using 90 samples by the NextSeq 300 cycles mid output kit v2.5. Because in all of the tested samples the amplicon sequencing approach showed a low coverage of 20 bp on the 5′-end of exon 4, this part was additionally sequenced by the Sanger sequencing method as described elsewhere^31^ with the use of a specific primer pair (Supplementary Table 1).

#### Biostatistical analysis of NGS data

The raw data gained from the amplicon sequencing was demultiplexed and converted into the .fastq format, which was then analysed by the same pipeline using the NextGENe software (Softgenetics), as described elsewhere^32^. The GRCh37 genome and NM_000458.2 reference transcript were used for the reads mapping and analysis. Only the samples with minimal coverage > 100 × and variants with variant allele frequency (VAF) > 10% were chosen for further evaluation. The identified variants were manually inspected using IGV (Broad Institute) and classified according to the mutation impact^32^. Only the mutations of class 3, 4 or 5 (variants of unknown significance, likely pathogenic or pathogenic, respectively) were reported.

#### HNF1B promoter methylation

For the purposes of methylation analysis, the DNA samples were subjected to bisulfite conversion DNA using the EZ DNA Methylation-Lightning Kit (Zymo Research, Irvine, CA, USA) according to the manufacturer’s instructions. The PCR amplification of both the methylated and unmethylated alleles was carried out using primers (Supplementary Table 1) which were designed with the software Methprimer (https://www.urogene.org/cgi-bin/methprimer/methprimer.cgi). The amplified promoter region of *HNF1B* covers 15 CpG islands and it is located − 457 to − 202 bp (GRCh37) before the HNF1B transcription start site (TSS).

Furthermore, this region includes the CpG island (chr17:36105517–36105518, GRCh37) the methylation of which is associated with a decreased HNF1B expression^33^, as well as the CpG islands (relative to the TSS: − 238, − 240, − 267) which, according to TCGA data, correlated the most with the expression (mRNA) of HNF1B in kidney tumours. In our settings we were able to detect at least 5% of methylated DNA by High Resolution Melting (HRM) Analysis of the amplified PCR products. Each run included the converted DNA samples and a series of 100%, 20%, 10%, 5 and 0% universally methylated DNA controls mixed with non-methylated DNA (Human HCT116 DKO Non-Methylated DNA and Human HCT116 DKO Methylated DNA; Zymo Research). The melting curves of the analysed samples were compared with the melting curves of the control mixes^34^.

## Results

### Immunohistochemical findings

**Figure 1:**
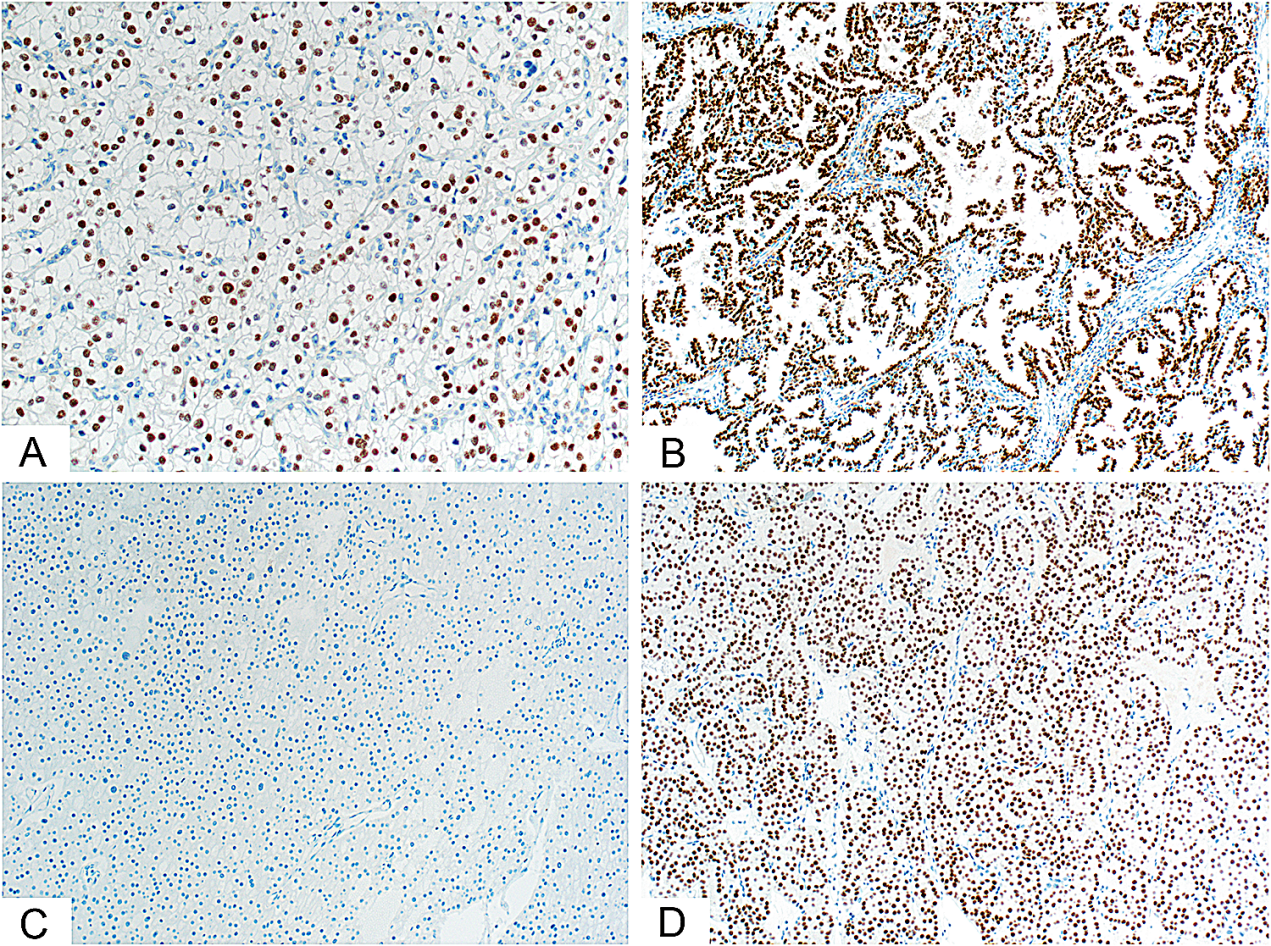
Immunohistochemical staining of the HNF1B expression in kidney lesions. (**A**) Predominantly moderate-to-strong nuclear expression in ccRCC (200 ×). (**B**) Strong, diffuse nuclear expression in papRCC (100 ×). (**C**) Complete lack of nuclear expression in chRCC (100 ×). (**D**) Moderate-to-strong diffuse nuclear expression in RO (100 ×).

The results of the IHC staining analysis revealed that there were significant differences in the presence and intensity of HNF1B expression among the four analysed subsets of kidney tumours (p < 0.001). The results of HNF1B expression for all of the tumour types are summarized in Table 1. In the subset of ccRCC, papRCC and RO, virtually all of the analysed tumour samples showed a certain level of homogenous, positive nuclear expression of HNF1B, while the subset of chRCC showed predominantly a complete negativity of the staining, with only two cases being weakly to moderately positive. Figure 1 shows representative examples of HNF1B expression in each of the assessed kidney tumour types.

**Figure 2:**
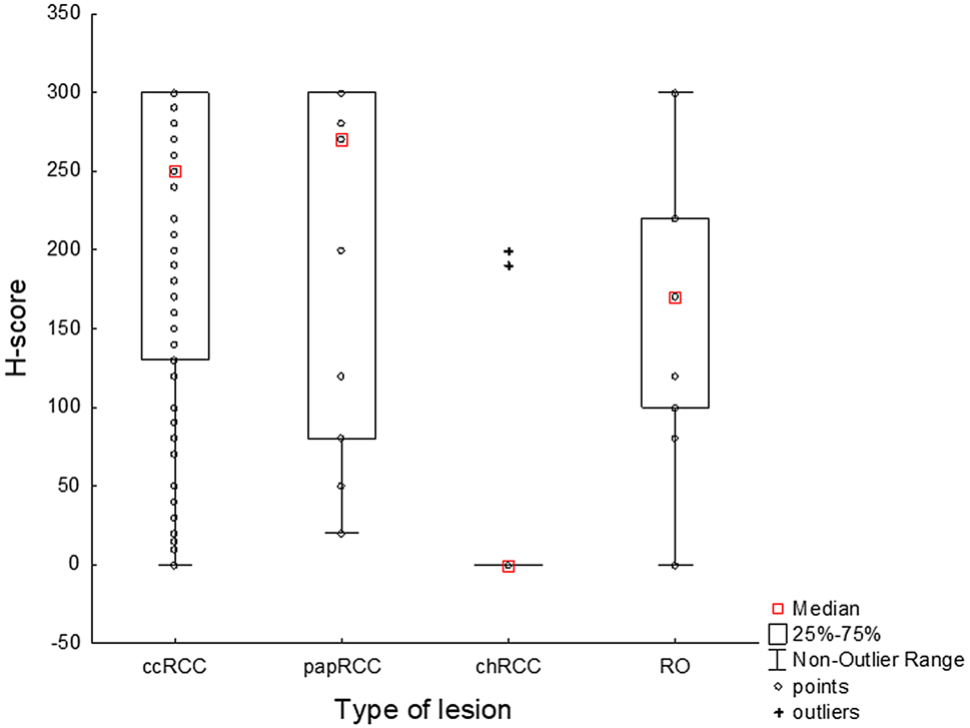
Variability of the expression of HNF1B (H-score) in relation to the type of lesion. Analysis based on 130 cases of RCC (ccRCC: n = 93, chRCC: n = 11, papRCC: n = 17, RO: n = 9). Kruskal–Wallis H-test: H (3,130) = 21.96, p < 0.001 (for the results of the post-hoc test, see the “Results” section). One point in the graph may represent more than one case.

The differences in expression of HNF1B (H-score) in relation to the type of kidney tumour are shown in Table 1 and Fig. 2 (also emphasizing the unbalanced sample size in each group). Subsequent post hoc test showed significant differences between the chRCC and ccRCC groups (p < 0.001), and also between the chRCC and papRCC groups (p < 0.001). No significant differences were detected between the RO and other groups.

**Figure 3:**
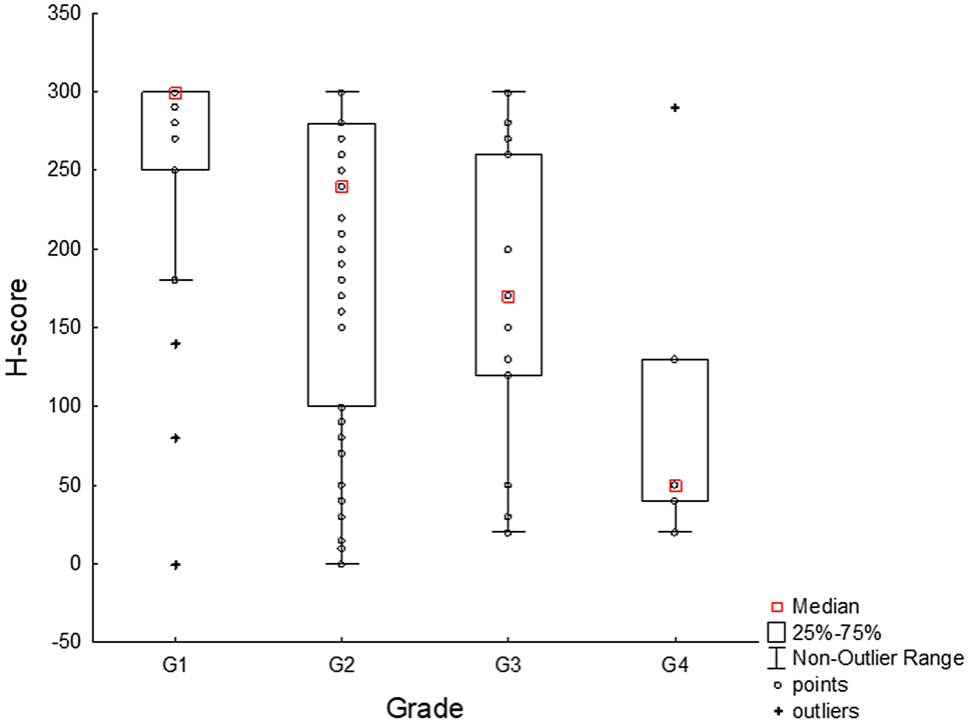
Variability of the expression of HNF1B (H-score) in relation to grade. Analysis based on 93 cases of ccRCC (G1: n = 22, G2: n = 53, G3: n = 15, G4: n = 5). Kruskal–Wallis H-test: H (3,93) = 14.50, p = 0.002 (for the results of the post-hoc test, see the “Results” section). One point in the graph may represent more than one case.

Given the unbalanced number of cases in the individual tumour subsets and their limited size, the association between HNF1B expression and clinicopathological characteristics was only analysed for the largest subset of ccRCC. The results showed that the expression of HNF1B was significantly correlated with tumour grade (p = 0.002), where a lower expression was associated with a higher tumour grade, with the following post-hoc intergroup differences: G1 vs. G2 (p = 0.032), G1 vs. G3 (p = 0.019), G1 vs. G4 (p = 0.025), G2 vs. G3 (p > 0.05), G2 vs. G4 (p > 0.05), G3 vs. G4 (p > 0.05) (Fig. 3). None of the other evaluated parameters (gender, age, T stage of the tumour, lymphovascular invasion, presence of metastases, recurrence, or presence of *HNF1B* SNP variants) showed any association at a significant level. However, when comparing the tumours of T1a and T1b stage (it would not be reasonable to analyse other T stages due to the low number of cases in each group), there was a significant difference in the H-score between these two stages, with T1a tumours showing a higher HNF1B expression than T1b tumours (pT1a: mean = 235.8, median = 280; pT1b: mean = 181.0, median = 185, p = 0.046; data not shown in Table 2).

### Genetic and epigenetic changes of the HNF1B gene

#### Mutation analysis

Mutation analysis was successfully performed on 53 samples of tumour tissue (39 ccRCC, 2 papRCC, 3 chrRCC, 9 oncocytomas) and 42 samples of corresponding healthy tissue. There were no class 3–5 mutations found in the *HNF1B* coding regions and their flanking areas in any of the analysed samples.

As a part of the mutation analysis we also focused on the role of two of the most commonly reported SNPs associated with an increased or decreased risk of certain cancers (especially prostate and endometrial carcinoma)—the rs4430796 and rs7527210 variants. No significant association was found for either of the two SNPs and HNF1B expression.

### Epigenetic analysis

The *HNF1B* promoter methylation analysis was performed on the total of 53 samples of tumour tissue and 39 samples of corresponding healthy tissue, which underwent the bisulphite DNA conversion. Promoter methylation was detected in 2/39 ccRCC (5.1%) and 1/9 (11.1%) RO. In all three of these cases the detected methylation was weak (5–10%, H-score of these tumours was 300 and 10 for the ccRCC, and 300 for the RO). Non-tumour tissue was available only for one of the methylation positive ccRCC tumour samples and no methylation was detected.

## Discussion

HNF1B is a transcription factor from the homeobox-containing family of transcription factors, which is known predominantly for its role in the development of endoderm-derived organs, with a special importance in the development of kidneys^35^. However, there is emerging evidence that it may also play an important role in the carcinogenesis of several different types of solid tumours. In this context, *HNF1B* is a master regulator gene which exerts its influence through maintaining active transcription or counteracting the silencing effect induced by mitotic chromatin condensation^36^. While reports about the significance and specific pathogenetic role of HNF1B in tumorigenesis are often ambiguous, there is a consistent finding of positive HNF1B expression reported in association with tumours of clear cell phenotype, given that over 90% of these tumours show predominantly strong levels of HNF1B positivity on a protein level^21, 37–40^.

We have performed a comprehensive, multi-level analysis of the *HNF1B* gene on several of the most common kidney tumour subtypes. The mutation analysis did not reveal the presence of any somatic or germline class 3–5 mutations in *HNF1B*. This finding is consistent with the literature, which shows that while there are over 100 reported germline mutations which usually manifest themselves in the form of rare kidney developmental disorders^10^ very little is known about somatic *HNF1B* mutations and their significance. However, there are a small number of case studies which state that HNF1B-renal disease is associated with a predisposition to chRCC development^18, 41^.

In contrast to somatic *HNF1B* mutations, much more information is available about the significance of *HNF1B* SNPs and their association with either an increased or decreased risk of the development of several cancers. In our sample set of ccRCC, we analysed the relationship between the two most commonly implicated SNPs mentioned in literature (rs4430796 and rs757210) and their HNF1B expression, but no correlation was found. In our study, the key events in *HNF1B* alterations are therefore probably mediated either by different mechanisms, or by the effects of rarer SNPs, which have not been included in the analysis.

**Figure 4:**
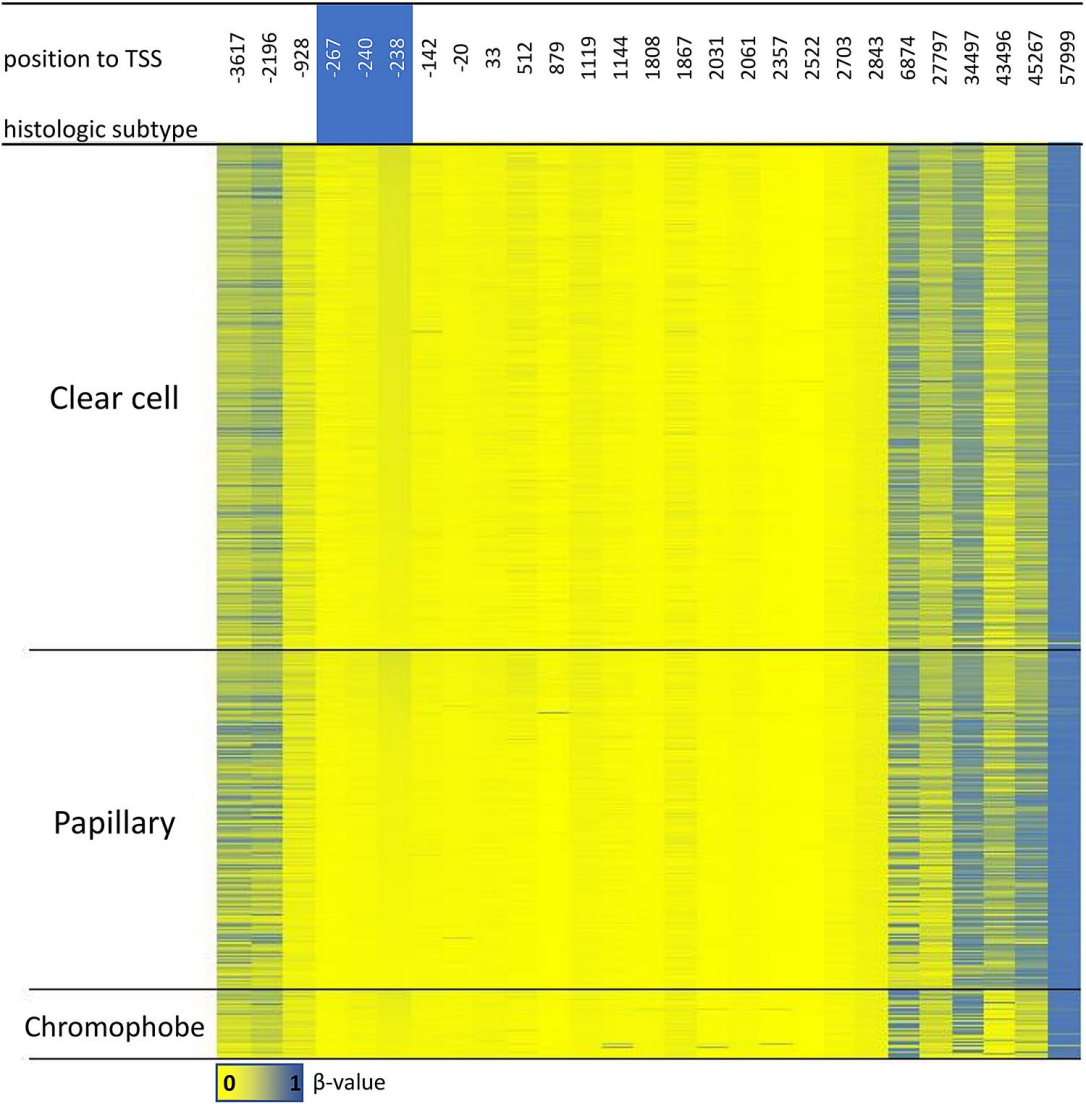
Methylation of the *HNF1B* gene in kidney tumours from TCGA database. The visualized data of methylation analysis of all the CpG islands of HNF1B *loci* was downloaded through Mexpress (https://mexpress.be, accessed January 2020). Each row corresponds to tumour tissue sample, each column represents one of the 27 analysed CpG islands, the number above the column indicates the position of the CpG island from the transcription start site (TSS) of the *HNF1B* gene. Three of the CpG islands (in the blue box) were also included in our promoter region analysis.

Epigenetic changes also play an important role in cancerogenesis as they influence gene expression and are frequently implicated in the development of cancer^39^. In mammalian cells, the most frequently occurring such alteration is DNA methylation, which is a reversible process and therefore represents a potential therapeutic target^42^. In our study, we found that promoter methylation was a rare event, only detected in 3 cases (2 ccRCCs, 1 RO), suggesting that there are other mechanisms aside from promoter methylation involved in the regulation of HNF1B expression. However, we were focused only on the CpG island area which correlates the most with HNF1B expression according to TCGA and literature^33^, so in order to fully discard the role of promoter methylation more inclusive analysis is needed. Our methylation results are in accordance with the data gained from TCGA, which shows methylation of the proximal promoter region in 2/480 of ccRCC, 1/321 papRCC and 0/66 chRCC (β value > 0.3 as a threshold for positive DNA methylation (Fig. 4)^43^.

In adult kidney parenchyma, a strong HNF1B nuclear expression is preserved in the tubular epithelia and the cells lining Bowmann´s capsule, while the glomerular structures and surrounding stromal tissues are negative^21^. When evaluating the immunohistochemical expression of HNF1B in our sample sets, there were significant differences among some of the four studied tumour types.

The lowest HNF1B expression was observed in the subset of chRCC, where it was either significantly low [weak positivity in 2/11 cases (18%)], or more commonly completely absent [complete negativity in 9/11 cases (82%)]. However, these results are gained from a limited sample set and a more extensive analysis of a larger cohort is needed to better visualize the level of HNF1B expression downregulation. Nonetheless, very similar results were reported in the study by Szponar et al., who in their sample set of 21 chRCC did not observe any nuclear positivity at all^23^. Similarly, another study examined 18 chRCC and found HNF1B to be underexpressed in 16/18 (89%) cases^44^. On a molecular level, chRCC is characterised by a specific pattern of aneuploidy with the loss of entire copies of chromosomes 1, 2, 6, 10, 13, and especially 17 (where *HNF1B* is located)^41^. It has been reported that in a majority of chRCC there is a downregulation of HNF1B expression on both the mRNA and protein levels, and the same study observed a strong correlation between the reduced expression of HNF1B and aneuploidy in chRCC patients^36^. The authors also point to the role of additional loss of *TP53*, which may allow the cells to escape senescence and death. The combined *HNF1B* loss and *TP53* mutations increase cell proliferation and aneuploidy, promoting an aggressive phenotype in chRCC^36^. In the setting of chRCC, HNF1B may therefore have a tumour suppressive effect.

In our study, the highest expression of HNF1B was observed in the subset of papRCC followed by ccRCC, despite the striking differences in the hypothesized origin and pathogenesis of these two types of cancers.

On a molecular level, papRCC is characterised by specific trisomies and tetrasomies, especially of chromosome 7 and 17, with the genes *MET* (7q31) and *HNF1B* being particularly implicated in the pathogenesis of papRCC. The amplification and overexpression of *MET* has been linked to impaired differentiation of affected cells, which is then followed by copy number gain or amplification of chromosome 17, leading to an overexpression of HNF1B^3, 23^. The resulting constitutive overexpression of HNF1B may further promote the proliferation of cells with delayed or arrested differentiation, which may be a part of the step-wise process of papRCC pathogenesis. Our results revealed that all the 17 papRCC showed some levels of HNF1B positivity, with most of the cases beingly strongly positive. Varying degrees of HNF1B positivity were also reported by Szponar et al., who observed strong nuclear HNF1B positivity in 38/67 (57%) papRCC in their sample set, as well as by Banyai et al., who observed a moderate IHC positivity in 47/76 (62%) of cases^3, 23^. Given that HNF1B is consistently reported as being overexpressed in adult tumours of embryonal origins (papRCC, but also mucinous tubular spindle cell carcinoma and metanephric adenoma), this overexpression may be a driver of papRCC development. In papRCC, HNF1B may therefore function as a protooncogene.

Contrary to papRCC, in the case of ccRCC the tumour origin has been attributed to the epithelial cells lining the proximal tubule of the kidney nephron. In our subset of ccRCC we observed varying levels of nuclear expression in almost all the examined cases [91/93 (98%)], which is in keeping with the observation that HNF1B expression is preserved, but variably attenuated in tumour tissues compared to normal kidney^45^. Preserved HNF1B expression was also reported by Wang et al., who observed it in 23/24 (96%) cases of ccRCC in their study^44^. Contrary to those results, Szponar et al. reported that in their study only scattered nuclear positivity of HNF1B was observed in 7/98 (7%) ccRCC^23^. In our work, the expression was significantly correlated with the grade of the tumour, where a lower expression was associated with a higher grade.

The association between decreased HNF1B expression and malignant potential of ccRCC was also studied by Buchner et al., who examined the expression of HNF1B in ccRCC metastases on an mRNA level and found that the downregulation of HNF1B in ccRCC was associated with tumour progression and poor prognosis, suggesting that HNF1B might be a useful prognostic factor when stratifying patients with metastatic ccRCC into prognostic groups^21^. The authors hypothesize that the inactivation of HNF1B expression results in the deregulation of the transcriptional network, which leads to tumorigenesis and tumour progression. As such, the role of HNF1B in ccRCC could therefore be in the form of a tumour suppressor. However, more data is needed to confirm that hypothesis, especially given that this result is in stark contrast to the other most common tumour with a clear cell phenotype, OCCC, because in OCCC the overexpression of HNF1B is a common phenomenon and has been linked to the development of this tumour^46^.

These results highlight the different natural history between papRCC and ccRCC, considering that the mutations and molecular pathways involved in their development are different. In ccRCC, the significance of HNF1B is not only in its potential use as a prognostic factor, but its potential therapeutic role is also being discussed. Given that the role of HNF1B in the pathogenesis of ccRCC seems to lie in its inactivation, leading to an impaired transcription network, it is possible that the reactivation of HNF1B and its signalling role could restore the dysfunctional network^21^.

Regarding the IHC analysis, the difference in HNF1B expression between the subset of chRCC and RO may be of particular interest for routine diagnostic practice. RO is the second most frequent benign renal tumour of the renal parenchyma and represents 3–7% of all renal lesions^47^. Among others, morphologically it is characterised by eosinophilic features, which may resemble the eosinophilic subtype of chRCC and the distinction between these two entities can represent a diagnostic challenge^48^. Given that these are tumours with crucially different biological behaviours, proper diagnosis is key for correct postoperative surveillance and for the prognosis of the patient. In our study, following the post-hoc tests the difference in HNF1B expression did not reach statistical significance (p = 0.080). To date, no single, reliable immunohistochemical marker of oncocytoma and chRCC has been described. There are several studies which focused on the possible role of HNF1B in differentiating between RO and chRCC, however, they are often performed on small sample sets and report conflicting results^23, 44, 49^.

In order to maximize the therapeutic effects of any chosen treatment, individualized prognostic stratification of patients is needed. Currently, the established prognostic parameters for ccRCC include the tumour histological type, pTNMG classification and the presence of necrosis and microscopic vascular invasion^24^. With the increasing use of targeted medicine, given its hypothesized different role in the development of different subtypes of RCC, the expression of HNF1B could potentially represent another prognostic factor enabling a better stratification of patients.

## Conclusion

RCC is comprised of a diverse spectrum of carcinoma subtypes with distinct morphology, molecular pathology, pathogenesis, and prognosis. Despite the advances in understanding the genetic aberrations behind the development of various RCC subtypes, the search for new prognostic markers, as well as potential therapeutic targets and differential diagnostic ancillary tests is constantly ongoing. HNF1B has already been well established as playing a crucial role in the development of the kidneys and as such could also be implicated in the pathogenesis of RCC. We have performed a comprehensive, multi-level analysis of HNF1B in several different kidney tumour types and found that the genetic and epigenetic analyses did not reveal any *HNF1B* mutations and only rare promoter methylation. The immunohistochemical analysis revealed that there are significant differences in HNF1B expression between the four studied tumour types. The different HNF1B expression suggests that in the setting of chRCC and ccRCC, HNF1B may be involved as a tumour suppressor, while in papRCC its role may be as a protooncogene. However, in order to determine the precise role of HNF1B in kidney tumour biology and its potential prognostic and therapeutic application, more studies on larger cohorts are needed.

## Supplementary information

Supplementary Table S1

## Data Availability

The source data are included in this article and its supplementary information file or are available from the corresponding author upon reasonable request.
