# Supplementary material for: Analysis of expression, epigenetic, and genetic changes of HNF1B in 130 kidney tumours
Source: Sci Rep. 2020 Oct 13;10:17151. doi: 10.1038/s41598-020-74059-z (PMC7555858; doi:10.1038/s41598-020-74059-z)
Supplement: Supplementary file 1 — Supplementary Table S1 [file 41598_2020_74059_MOESM1_ESM.docx]

**Analysis of expression, epigenetic, and genetic changes of HNF1B in 130 kidney tumours.**

Running title: HNF1B in kidney cancer.

*Michaela Bártů^1^, Jan Hojný^1^, Nikola Hájková^1^, Romana Michálková^1^, Eva Krkavcová^1^, Ladislav Hadravský^1^, Lenka Kleissnerová^1^, Bui Quang Hiep^1^, Ivana Stružinská^1^, Kristýna Němejcová^1^, Otakar Čapoun^2^, Monika Šlemendová^2^, *Pavel Dundr^1^

^1^Institute of Pathology, First Faculty of Medicine, Charles University and General University Hospital in Prague, 12800 Prague, Czech Republic,

^2^Department of Urology, General University Hospital in Prague, 12808 Prague, Czech Republic

Corresponding authors:

Pavel Dundr, M.D., Ph.D.

Institute of Pathology, First Faculty of Medicine, Charles University and General University Hospital in Prague, Studnickova 2, 12800 Prague 2, Czech Republic.

Email: [pavel.dundr@vfn.cz](mailto:pavel.dundr@vfn.cz)

Michaela Bártů, M.D.

Institute of Pathology, First Faculty of Medicine, Charles University and General University Hospital in Prague, Studnickova 2, 12800 Prague 2, Czech Republic.

Email: [michaela.bartu@vfn.cz](mailto:michaela.bartu@vfn.cz)

**Supplementary Table S1.** List of gene-specific primers used for HNF1B mutation analysis with universal adaptor sequences (red) by amplicon NGS.
